# Supplementary material for: Synthesizing Stakeholders Perspectives on Online Psychological Interventions to Improve the Mental Health of the Italian Population during the COVID-19 Pandemic: An Online Survey Study
Source: Int J Environ Res Public Health. 2022 Jun 8;19(12):7008. doi: 10.3390/ijerph19127008 (PMC9222987; doi:10.3390/ijerph19127008)
Supplement: Supplementary file 1 [file ijerph-19-07008-s001.zip › ijerph-1704009-supplementary.pdf]

Table S1 – Characteristics and baseline information of the sample

	n	%
**Gender**		
Male	309	30.2
Female	715	69.8
**Age (range)**		
Young adults (18-40)	553	54.2
Adults (41-65)	388	38.0
Elderly (66-89)	79	7.7
**Education**		
Primary School	8	0.8
Middle School	118	11.6
High School	290	28.3
Degree	431	42.1
Master	92	9.0
Ph.D.	84	8.2
**Job position (before the COVID-19 pandemic)**		
Student	140	13.7
Part-time employee	108	10.6
Full-time employee	396	38.7
Freelance	240	23.4
Housewife	40	3.9
Unemployed	28	2.7
Retired	72	7.0
**Change in job position due to the COVID-19 pandemic**		
Yes	243	23.7
No	777	75.9
Missed	4	0.4
**Current job position**		
Unchanged	181	17.7
Smart working/Distance learning	341	33.3

Layoffs	60	5.9
Discontinued activity (if owner)	16	1.6
Unemployed	100	9.8
Retired	80	7.8
Mixed mode (from home and in presence)	68	6.7
Maternity	33	3.2
Reduction of working hours	20	2.0
Missed	124	12.0
**Infected by the virus**		
Yes	144	14.1
No	880	85.9
**Loved one(s) infected by the virus**		
Yes	555	54.2
No	469	45.8
